# From the Clinical Problem to the Basic Research—Co-Culture Models of Osteoblasts and Osteoclasts

**DOI:** 10.3390/ijms19082284

**Published:** 2018-08-03

**Authors:** Sheng Zhu, Sabrina Ehnert, Marc Rouß, Victor Häussling, Romina H. Aspera-Werz, Tao Chen, Andreas K. Nussler

**Affiliations:** Department of Trauma and Reconstructive Surgery, Siegfried Weller Institute for Trauma Research, Eberhard Karls University Tuebingen, BG Trauma Center Tuebingen, 72076 Tuebingen, Germany; zhusheng8686@gmail.com (S.Z.); sabrina.ehnert@gmail.com (S.E.); m.ruoss@hotmail.de (M.R.); victor.haeussling@hotmail.de (V.H.); rominaaspera@hotmail.com (R.H.A.-W.); zzuchentao@yahoo.com (T.C.)

**Keywords:** co-culture, osteoblasts, osteoclasts, 2D cultures, 3D scaffolds, bone metabolism

## Abstract

Bone tissue undergoes constant remodeling and healing when fracture happens, in order to ensure its structural integrity. In order to better understand open biological and clinical questions linked to various bone diseases, bone cell co-culture technology is believed to shed some light into the dark. Osteoblasts/osteocytes and osteoclasts dominate the metabolism of bone by a multitude of connections. Therefore, it is widely accepted that a constant improvement of co-culture models with both cell types cultured on a 3D scaffold, is aimed to mimic an in vivo environment as closely as possible. Although in recent years a considerable knowledge of bone co-culture models has been accumulated, there are still many open questions. We here try to summarize the actual knowledge and address open questions.

## 1. Introduction

Bone healing problems have existed for as long as human beings, but were for a long time not considered as very important, since they were mainly seen in the elderly. However, nowadays a dramatic worldwide demographic change towards an aging society along with a high increase of metabolic diseases, such as diabetes mellitus type 2, forces society to tackle the gaps in knowledge about bone disorders, such as osteoporosis. Several studies confirmed that a number of comorbidities, such as smoking, kidney diseases, diabetes or nonsteroidal anti-inflammatory drug (NSAID) abuse, increase the complication rate of fracture healing [1,2,3,4,5,6]. Moreover, we and others identified that malnutrition is another important risk factor clearly linked to delayed bone healing and delayed patient discharge from the hospital [7,8,9].

Since our knowledge about osteosynthesis in general is still limited, researchers around the world try to overcome this deficit by using in vitro cell cultures. Some of these cell cultures have been further developed by mimicking various comorbidities [10,11]. To address all of the above, the following questions might be of importance:

- Which cell type(s) should be used?
- Single mono-culture model or a co-culture model?
- Which cell culture type should be used?
  o two dimensional (2D) or three dimensional (3D)?
  o static or dynamic?
- In case of 3D cultures, which matrix should be used?
- What are the molecular and functional markers to validate possible changes in the applied co-culture models?
- How can co-culture models be normalized to study cell specific changes within the co-culture models?

Today, it is widely accepted that an imbalance of osteoblasts and osteoclasts in several bone disorders leads to increased fragility and decreased bone strength [12,13,14]. The main cell types to study bone disorders are osteoblasts and osteoclasts. In order to study processes and dynamics of how osteoblasts and osteoclasts are interacting in certain bone diseases, different cell culture models were developed addressing these open questions. However, one of the major hurdles is the investigation of the cellular interactions in vitro [15]. While analyses of secreted factors can easily be done, investigating direct cell-cell or cell-matrix interactions requires more complex cell culture models. However, the increasing complexity often hampers the use of established/standard methods.

## 2. Osteoblast and Osteoclast Interaction

The most common cell types for studying bone diseases in vitro are osteoclasts, which resorb the bone tissue, and osteoblasts, which rebuild bone tissue [16,17]. Osteoblasts, and the mature osteocytes, built the solid foundation to achieve and maintain normal bone mass [18], while osteoclasts are regarded as the only cell type responsible for bone resorption [19]. As the most crucial cells during osteogenesis and remodeling, these cells do not act independent of each other; several communication pathways have been identified until now [20]. Cells of the osteoblast lineage developed an internal regulatory mechanism, e.g., osteocytes that start secreting sclerostin (SOST) as negative feedback for osteoblasts, when the mineral content of the surrounding is getting too high. Direct contact of osteoblasts/osteocytes and osteoclasts allows membrane-bound ligands and receptors to interact and initiate intracellular signaling. Moreover, gap junctions can be formed between the two cell types, allowing the passage of small water-soluble molecules related to proliferation and differentiation of cells, like calcium ions, cyclic adenosine monophosphate (cAMP), IP_3_, saccharides, amino acids, nucleotides, and vitamins [21,22,23]. Among cells, paracrine pathways are regarded as important communication systems, meaning that either cell type can secrete paracrine factors that act on neighboring cells via diffusion. For example, pre-osteoblasts synthesize the receptor activator of nuclear factor-κb ligand (RANKL) and osteoprotegerin (OPG), which regulate the differentiation and formation of osteoclasts. In return, sphingosine-1-phosphate (S1P), platelet derived growth factor (PDGF) or hepatic growth factor (HGF) secreted by osteoclasts have effects on activation of osteoblasts [24,25,26]; moreover, factors like transforming growth factor beta (TGF-β), bone morphogenetic proteins (BMP), insulin-like growth factor (IGF), collagen, and osteocalcin (OC) secreted and incorporated into the bone matrix by osteoblasts, may get liberated and in some cases even activated by osteoclasts [27]. These examples clearly show that bone remodeling is based on communication and regulated by the balance between osteoclasts and osteoblasts, which also varies at different stages of differentiation [28,29,30]. From all the aspects above, several molecules mediate communication between osteoclast and osteoblast lineages. Therefore, it is not sufficient to study osteoblasts and osteoclasts separately, when trying to decipher mechanisms underlying the various bone disorders [31,32].

In recent years, by applying new bio- and cell engineering tools, many new and important discoveries were drawn from co-cultures, shedding light into the osteoblast-osteoclast interplay. Simon et al. suggested that galectin-3 (a member of the β-galactoside-binding lectin family), secreted by both osteoblasts and osteoclasts, acts as an independent regulator of osteoclastogenesis [33]. Cai et al. found that the downstream of kinase-3 could negatively regulate osteoclastogenesis and enhance osteoblastogenesis by affecting M-CSF and RANKL [34]. C-Mpl, the receptor for thrombopoietin (THPO), has been considered to enhance bone formation. C-Mpl is expressed on both osteoblasts and osteoclasts and increases the number of osteoblasts while decreasing at the same time the number of osteoclasts, without affecting the major signaling pathways of M-CSF/OPG/RANKL and EphrinB2-EphB2/B4 [35]. Kang illustrated how IL-23 regulates the proliferation and activation of osteoclasts [36].

**Figure 1:**
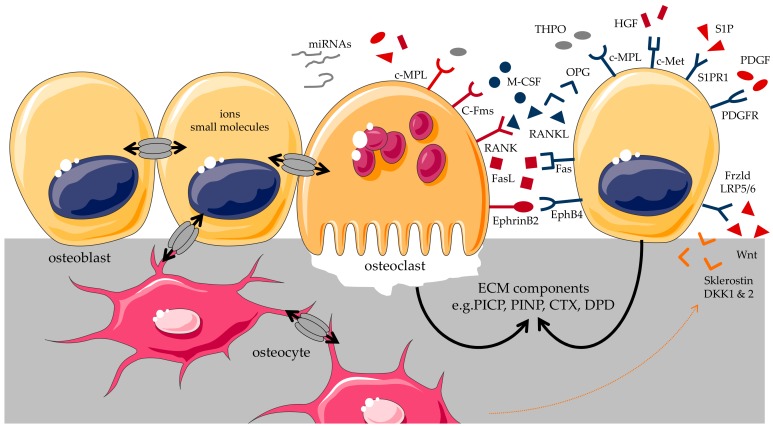
Communication between osteoblast and osteoclast through cell-cell contact (gap junction or EphB4-ephrinB2), paracrine factors (e.g.; M-CSF: macrophage colony stimulating factor, OPG: Osteoprotegerin, RANKL: receptor activator of nuclear factor-kb ligand, THPO: thrombopoietin, S1P: sphingosine-1-phosphate, PDGF: platelet derived growth factor) and their interaction via the bone matrix [21,22,24,26,27,32]. HGF: hepatic growth factor; PICP: procollagen type I carboxy-terminal propeptide; PINP: procollagen type I N-terminal propeptide; CTX: collagen type 1 C-telopeptide; DPD: deoxypyridinoline; DKK1 & 2: dickkopf 1 & 2. Graphical components were obtained from https://smart.servier.com/.

Apart from their role in bone resorption, osteoclasts have recently attracted more attention for directly modifying osteoblasts’ function. For example, soluble factors such as Sphk1, Wnt10b and BMP6 are secreted by osteoclasts and have different effects on osteoblasts [37]. Furthermore, exosome-mediated transfer of genetic information may represent a novel communication for osteoblasts and osteoclasts/osteocytes under certain physiological conditions. Thus, exosome-shuttered and circulating miRNAs are likely to add a missing piece to the bone cells communication [38] (Figure 1).

Basically, the balance of formation and resorption has a critical influence on bone mass and strength throughout a life with a subsequent period of stability followed by continuous decline during aging, with an increased osteoclast activity and a declined osteoblast activity, as seen in primary osteoporosis [39]. Therefore, it is vital to establish perfect biological cell culture models to study the process and pathogenesis of bone remodeling in various bone disorders.

## 3. Co-Culture Models

Obviously, to gain better insight into the communication between osteoblasts and osteoclasts, co-culture models are mandatory. In general, co-culture models can be defined as simultaneous cultivation of two or more populations of cells in a way that allows direct or indirect communication/contact with each other [40]. Forty years ago, the first co-culture study was published, which showed that interactions between rat granulosa cells and myocardial cells happen via gap junctions [41]. Since then many other studies, using co-cultures, followed [42,43,44,45]. Traditionally, co-culture models were carried out in 2D. This way, many methods established for 2D single cell cultures could be easily adapted to the co-cultures [46]. Until now, these 2D co-culture models can be categorized into two types: those which examine the effects of direct cell–cell interaction, and those which focus on paracrine factors and signaling connections. The first requires seeding of the different cell types in one common culture reservoir with a defined cell-cell ratio [47,48]. The second, indirect co-culture model requires some kind of permeable barrier that separates the cell types from each other in the culture reservoir, but allows exchange of secreted factors between them. This permeable barrier can either be a removable permeable divider [49] or a transwell system [50]. When the effect of one special cell type on another cell type is investigated without the physiological feedback, transfer of conditioned medium from one cell type to another is a feasible option [51,52]. In the same line, one type of cells can be seeded onto an extracellular matrix (ECM) that has been produced by another type of cells [53].

The described 2D co-culture models have several advantages but also some limitations. Using well established methods for 2D mono-cultures, it is fairly easy to observe functional changes within the co-culture system. Without a physical barrier between the different cell types, fewer methods are available, that allow investigating physiological changes of one of the involved cell types. However, based on the nature of the physical barrier, no direct cell-cell interactions can be analyzed. For the overview see Table 1.

**Figure 2:**
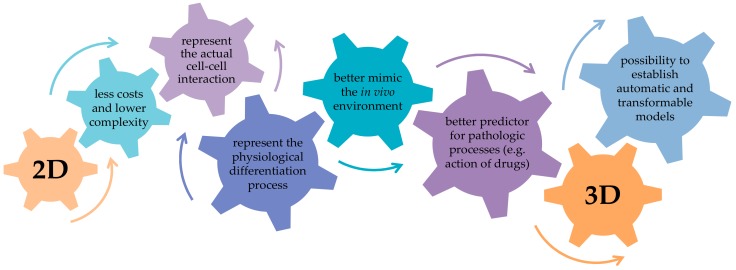
Conventional 2D cultures convince with a simple handling and a large variety of analytical methods available. However, by introducing co-cultures of cells into a 3D environment, functionality of the cells can be significantly improved, as it better mimics the in vivo situation. Further dynamization, e.g., perfusion or application of mechanical loads, can be included considering that with increasing complexity of the system, the use of established analytical methods becomes limited.

When conventional 2D co-culture systems are used to demonstrate the responsiveness between cells, one has to keep in mind that these models still poorly mimic the conditions in the living organism [61]. Since 1968, when the concept of 3D cell cultures was first presented [62], many researchers benefited from 3D cultures. The main idea of all 3D models is to represent the natural environment found in vivo as well as possible (Figure 2). This indicates that 3D systems allow the cells not only to survive on the 3D matrix, but it also allows for their proliferation and infiltration into it. Therefore, the used 3D matrix should represent the material and structure of the natural environment as closely as possible. With an optimal combination of mechanical properties, geometry and surface chemistry, the 3D system should allow good cell adherence, sufficient supply of oxygen, and adequate diffusion of nutrients and waste products.

One of the first 3D cultures reported were in hydrogels that can be defined as water-swollen networks of polymers. The simplest way to culture cell in a hydrogel is to embed the cells inside the hydrogels by mixing the cell solution with the hydrogel solution prior to gel formation. A bit more time consuming is the so-called sandwich technique, where cells are seeded onto a hydrogel layer and then, after cell attachment, are covered with a second hydrogel layer (a method frequently used for hepatocyte cultures [63]). Compared to classical 2D cell cultures, hydrogels allow the cells to better preserve their physiological shape and function, thus mimicking better the in vivo environment [64,65,66,67]. Hanging drop technique and 3D petri dish are both based on the principle of cell aggregation. With these methods, cells aggregate within drops of medium, that are maintained with minimal evaporation and without spreading [68,69,70]. Three-dimensional scaffolds, traditionally made of polymeric biomaterials, have the advantage of providing a structural support for cell attachment and tissue development. In the past 50 years, numerous natural and synthetic scaffolds have been developed for different specific cell culture setups [71,72,73]. For an overview see Table 2. For bone cultures, often 3D scaffolds, containing collagen and/or hydroxyapatite, are used.

The knowledge that 3D culture conditions and their dynamization may improve cellular function is well established. Novel co-culture systems are frequently carried out in a 3D environment, often also with active perfusion and/or other mechanical stimulation, which adds complexity to the model. With these adaptations, novel 3D cultures can mimic the in vivo situation much better [76]. However, with increasing complexity of the model, we face the limitations regarding the analytical methods. For example, the requirement for larger volumes of medium with (dynamic) 3D systems alters the nutrient supply, dilutes waste products and metabolites, and changes the oxygen supply. For an overview see Table 2.

As the demand for cell culture techniques is constantly increasing, more innovative and complex 3D co-culture models have been developed. For instance, tissue roll for the analysis of cellular environment and response (TRACER) is a proper 3D tumor model platform that enables collection and analysis of cells [77]. The main advantage of these further developed dynamic 3D culture models is long term cell analysis as compared to short term static models [74,75,78]. Despite all technical development, the present in vitro 3D co-culture model cannot completely represent the in vivo situation. To represent different pathophysiologic conditions (e.g., metabolic bone diseases), animal models are still the method of choice. Animals have the big advantage of being a system with all consisting cell types involved under optimal natural culture conditions [79]. However, besides ethical considerations, it is hard to clearly attribute which cell might be responsible for a specific condition. Furthermore, one has to keep in mind, that rodents with their short life span often show an altered cell metabolism, not only visible in drug metabolism (cytochrome P450 enzymes) but also in blood clotting, immune responses, etc. [80] Thus, while animal models reflect the whole complex state of bone regeneration better [81,82], the main advantage of dynamic 3D co-culture models is their use of specific (human) cell types for the verification of certain hypotheses.

## 4. Co-Cultures of Osteoblasts and Osteoclasts

Co-culture systems are of great importance in systems biology. As osteoblasts/osteocytes and osteoclasts affect each other via different complex interactions, diverse co-cultures combining osteoblasts/osteocytes and osteoclasts have been developed in recent years [83].

### 4.1. Selection of Culture Models

Co-culture models have numerous categories focusing on different research targets; therefore, it is fundamental to match co-culture models with the research purpose [84]. In 2D systems, both osteoblasts and osteoclasts are frequently well-differentiated and in direct contact with each other. In indirect co-cultures like transwells, osteoblasts and osteoclasts exclusively communicate by soluble factors in the medium. However, the best model to be established would be a multi-dimensional co-culture model that mimics the in vivo environment including perfusion and even biomechanical properties.

Based on current research on multi-dimensional co-culture models, 3D scaffolds are the most studied and developed model among research on the skeleton system due to the superior compatibility and diversity. Scaffolds shall be able to optimally mimic the bone through their mechanical and chemical properties.

In the past years, many laboratories have attempted to develop dynamic perfusion bioreactors to stimulate cells in a flow-dependent manner to further mimic the in vivo environment [85,86,87]. Recently, a vascularized 3D bone remodeling model has been developed, in which multiple cell types, including human umbilical endothelial cells, bone marrow mesenchymal stem cells (B-MSCs), osteoblasts and osteoclasts could exert their own functions to mimic the in vivo environment [88].

Basically, a simple 2D cell culture model with primary human cells is sufficient for a first establishment of an in vitro test system. Later on, more complex 3D systems integrating different cell types, fluidics, and mechanics should be established to gain deeper insight into the bone remodeling process or pathological state [89,90].

### 4.2. Requirements for the 3D Matrix Used for Culturing Bone Cells

For 3D culture systems, the selected matrix should have a structure resembling the bone; highly porous with interconnected pores, and support both cell attachment and proliferation. Furthermore, it is necessary that scaffolds have a good structural integrity to meet the requirements for mechanical properties of native tissue and allow transport of nutrients and waste products. Finally, it is desirable that scaffolds allow and support extracellular matrix (ECM) formation, but they shall not release substances harmful to the cells [91].

For each tissue, there are specific properties that should be met to support the optimal cell growth. An important parameter is the pore size. The average pore diameter of the decellularized cancellous bone is in a range of 389.3 ± 134.9 μm [92]. Several studies have shown that a pore size between 300 and 500 μM is necessary for the adhesion, differentiation and growth of osteoblasts [93,94]. In one study, it was also shown that with a pore size below 200 μM, bone formation does not occur [94]. The porosity of the matrix also plays an important role in bone cell scaffolds. Porous areas of the bone allow vascularization, cellular infiltration, proliferation and matrix deposition [95]. Another important feature of scaffold architecture is the degree of interconnectivity between the pores. Scaffolds, which have a highly interconnected architecture, promote tissue growth [96]. It has been shown that the interconnectivity of scaffolds has a direct effect on MSC signaling and differentiation, and subsequently on the morphology of bone formation [97]. In addition, the matrix stiffness is an important point that influences the osteogenic differentiation of MSCs towards osteoblasts, which preferentially occurs between 11–40 kPa [97,98].

In addition, RGD peptides play an important role in the scaffold development. Synthetic RGD peptides are able to enhance the cell response of the scaffolds with increasing mechanical and structural properties but a limited bioactivity. In addition, it is possible to tightly control the density, patterning, structure, and orientation of these peptide matrix molecules [99]. Greiner and co-workers showed that zoledronic acid incorporated in a poly(d,l-lactide) implant coating improves co-cultures of osteoblasts and osteoclasts [100].

Beside functionalizing the existing scaffolds, more novel scaffolds have been developed in recent years: the research group of Gelinsky and co-workers used synthetic nanosilicate clay, called Laponite, to build up scaffolds, using the extrusion-based 3D plotting method with highly viscous, high density collagen dispersion for experimental skeleton research application [101,102]. With a novel rotational co-culture, Clark et al. have generated a large, three-dimensional mineralized tissue mixed with primary osteoblast and osteoclast precursor cells of human origin [103]. Bernhardt et al. developed a material made of mineralized collagen I that mimics extracellular bone matrix and might be used for co-cultures of osteoblasts and osteoclasts [104]. A bone substitution material made of the three components—silica, fibrillar bovine collagen, and hydroxyapatite was used by Heinemann et al. to establish the co-culture model of human B-MSCs and human mononuclear cells [105]. Lutter et al. presented a naturally produced osteoblast derived ECM, which can be used as functional, easy-to-use and inexpensive in vitro test system to analyze bone resorption [106]. Moreover, mechanical stimulation influences the communication between osteoblasts and osteoclasts [107,108], which should be considered in future co-culture 3D scaffold designs.

### 4.3. Selection of Cells

The selection of appropriate cell populations in co-cultures should be deliberated for the reason that different cell types have their own physiological and pathological characteristics, which help to understand cell-cell interactions and identify potential therapeutic targets. In general, co-cultures employing primary osteoblasts and monocyte derived osteoclasts should be used for long-term observations, because of no cancerous or genetically transformed characteristics and its ability to represent the natural donor differences. So, primary cells are the so-called golden standard for cell culture [109]. Primary human osteoblasts should be the cells of choice to be used in all areas of in vitro bone biology research. However, human mesenchymal stem/stromal cells (MSCs) and human peripheral blood monocytes (PBMCs) are a good alternative for the co-culture systems, which are targeted at the interaction between osteoblast and osteoclast, avoiding the use of external supplements to induce the appropriate differentiation [110]. MSCs have the property to differentiate into different cell types, like osteoblasts, chondrocytes and adipocytes [111]. Therefore, a suitable microenvironment is required to induce the osteogenic differentiation of the MSCs and to lead to the formation of bone tissue [112]. MSCs can be isolated from several tissues, like bone marrow, adipose tissue or umbilical cord. Actually, bone marrow derived MSCs are the most frequently used stem cells today, showing high osteogenic capacity. Nevertheless, MSCs derived from adipose tissue (Ad-MSCs) could be a better alternative due to the easy access and availability of the tissue. MSCs are an interesting choice for co-culture models, studying the process of osteogenesis [59,113]. In the past years, human induced pluripotent stem cells (hiPSCs) have been used to differentiate into osteoblast and/or osteoclast precursors [114,115]. Although until now hiPSCs have been mostly tested to replace MSCs for bone regeneration in vitro [116,117], the cells might be used for future tissue engineering of (dynamic) 3D cultures. With the advantage that patient specific cells can be generated using hiPSCs, one has to keep in mind that the efficiency of the procedure might vary from donor to donor. Furthermore, when attempting a co-culture, the different differentiation protocols need to be synchronized or only differentiated cells can be combined.

Other primary cells, especially rodent cells, are useful for addressing the shortcomings of human cells, including the problems regarding donor age and sex differences [118]. However, animal-derived primary cells cannot represent the actual in vivo situation. Both human and animal primary cells also have limitations due to their slow proliferation and long doubling time. Moreover, after two or three cell passages, primary cells are frequently not able to divide any longer [119,120]. Hence, due to the limitation in amount and lack of reproducibility of primary cells, osteogenic cell lines have been used in most cell culture models due to their ease of handling, infinite cell numbers and stable phenotype with no need for cell isolation. Osteogenic cell lines have been widely used to detect the mechanism of cell differentiation, the function of cytokines and hormones, pathology of bone disease and new drug screenings [121]. In the specific experiment, it is important to characterize the compatibility of different cell lines and their seeding protocols in order to meet the purpose of model setup (Table 3). For example, when a certain cell line requires a specific factor for their function this might affect the order of the cell seeding. As an example, myeolytic cell lines, which might be used as precursors for osteoclasts require co-factors (e.g., PMA) for cell adherence [122]. Thus, when plating the osteoblastic cells first it has to be guaranteed that this factor does not affect their function. If this is the case, the osteoblastic cells have to be plated after the myeolytic cells (osteoclast precursors), when a complete removal of the disturbing factor is guaranteed [123,124]. When the exposure to possible disturbing factors cannot be circumvented by specific seeding orders, the culture media will have to be modified.

### 4.4. Selection of Medium

For co-culture models, a variety of basic media exist. Due to different components and concentrations of additives, results may vary, depending on the type of medium used. Except for mixing media of both cell types, recent research focuses on developing novel media with different supplements for co-cultures of osteoblasts and osteoclasts [114,126], as the classically used supplements might interfere with the other cell type.

As an example, in classical mono cell cultures osteoblasts require dexamethasone to induce their maturation. However, the function of mononuclear cells as precursors for osteoclast cultures gets inhibited by this supplement.

In co-culture systems, many supplements used in the respective mono-cultures might be no longer necessary, as the additional cell type might provide the factor. For example, in the classical mono cell culture, osteoclasts are derived from mononuclear cells by addition of a defined cytokine cocktail, containing M-CSF and RANKL. Both factors are normally produced by osteoblasts. Thus, in a co-culture approach, no additional cytokines should be needed. Simply by using the conditioned medium of differentiating primary osteoblasts, mononuclear cells can be differentiated into osteoclastic cells [51,52]. On the other hand, to avoid diverse supplements from interfering with the differentiation process of the other cell type in co-culture, Schulze presented a direct co-culture model of human B-MSCs and PBMCs without exogenous cytokines, which is suitable to mimic bone-remodeling [110].

## 5. Characterization and Normalization of Different Cell Types in Co-Culture Systems

It is widely accepted that osteoblasts and osteoclasts affect each other during differentiation by numerous factors. For classical 2D cultures, a great variety of methods are available that allow the characterization of osteoblastic and osteoclastic cells during their different phases of differentiation and maturation. However, when the culture systems become more complex, some of these established methods show limitations (Table 4).

Matrices in 3D culture systems are often not transparent. Thus, classical stainings (e.g., van Gieson, Alizarin Red, von Kossa, AP or TRAP staining) that are based on transmission microscopy cannot be applied. In case of AP or TRAP staining, the methods might be modified by replacing the conventional colorimetric substrate by a fluorogenic substrate. This way AP and TRAP positive cells can be detected at the surface of the 3D matrix [127]. However, to look deeper into the 3D matrices their sectioning/slicing is required. There are various possibilities available to do so, e.g., a microtome, a cryotome, a vibratome or a guided diamond blade saw. However, their use often requires the fixation and the embedding of the tissue. Porous structures tend to entrap air during the embedding procedure, which destabilizes the material during the cutting process. Furthermore, the fixation and embedding procedure impairs the use of staining methods that depend on the activity of cellular enzymes, e.g., AP and TRAP staining. Moreover, in the case of bone tissue, the dense mineralization might be too hard for most of the cutting devices. Thus, the tissues need to be demineralized first, which limits the analysis of the mineralized inorganic matrix.

When factors are secreted into the culture supernatant, their detection can often be done by the established methods, often based on enzyme linked immunosorbent assays (ELISA) or zymography. These methods face limitations, like for example when the target becomes too diluted, which might occur when the culture volume increases. Although static 3D cultures require larger volumes, the use of 3D matrices provide a much larger surface area than 2D cultures, thus the ratio of cells to medium usually increases in these cultures. However, when a perfusion is applied this might dramatically change.

Although the use of 3D cultures limits the use of several methods, it also opens up new possibilities. Three-dimensional matrices that are used for bone cell cultures are expected to change their mineral content, thus radiological methods can be used to detect changes in the formed mineralized matrix. In addition, the formation of organic and inorganic matrices affects the stiffness of the scaffolds, which can be physically measured by compression tests or atomic force microscopy (Table 4).

**Figure 3:**
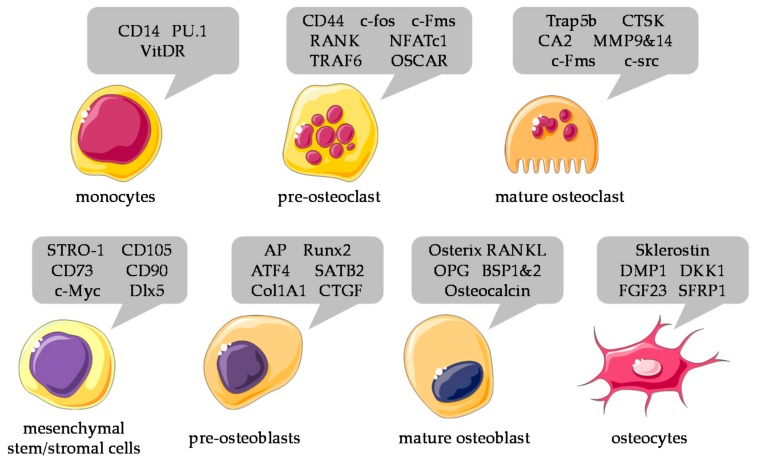
Numerous molecular markers can be detected in both osteoblast and osteoclast during differentiation; the figure above summarizes the most commonly used markers, including surface markers (vitamin D receptor (VitDR), osteoclast-associated receptor (OSCAR), STRO-1, or cluster of differentiation (CD)14, 44, 73, 90, 105)), cell specific proteins/enzymes (Trap5b, CTSK, CA2, MMP9&14, AP, Col1A1, CTGF, RANKL, OPG, BSP1&2, Osteocalcin, Sklerostin, DKK1, FGF23), transcription factors (PU.1, c-fos, Runx2, ATF4, SATB2, or Osterix), and other regulatory proteins (c-Fms, NFATc1, TRAF6, c-src, c-Myc, Dlx5, DMP1, SFRP1) Graphical components were obtained from https://smart.servier.com/.

To investigate the differentiation process of osteoblasts and osteoclasts, often expression of molecular markers is analyzed. For the different stages of differentiation of osteoblastic and osteoclastic cells, a large panel of molecular markers is available (Figure 3). However, one has to consider several things. Many 3D matrices impede the required RNA or protein isolation methods. In this case, it is often sufficient to adapt the isolation methods, e.g., with an additional cleaning step, to provide an adequate amount and purity of the samples. Different cell types used in co-culture systems raise another problem. The established normalization methods, e.g., mitochondrial activity, expression of a house keeper, DNA or protein content will not differentiate between the different cell types.

In this case, again fluorogenic staining can help. To visualize every single cell type, Schmid et al. 2018 used vibrant cell labeling (Gibco), where monocytes were stained by DiO (3,3'-dioctadecyloxacarbocyanine perchlorate/green) and MSCs by Dil (red) [161]. This allowed them to see the distribution as well as the amount of osteoblasts and osteoclasts in a 3D co-culture system, with a limited depth from the surface.

Immunofluorescence staining provides another method to normalize the different cell types on a matrix and also allows determining specific cell markers. Schmid F. et al., 2018 used f-actin fluorophore, vinculin and cathepsin-K as well as CD51/61-antibodies to visualize osteoclast-like cells [161]. Therefore, it is possible to visualize cells on the surface of the scaffold and normalize by staining. However, a further challenge is to analyze and normalize cells within a 3D-scaffold.

## 6. Recent Developments and Future Challenges

With an increasing incidence of osteoporosis [162,163], and metabolic disease-associated fractures, the socio-economic pressure will demand more and more answers to unsolved questions. Therefore, highly sophisticated bone cell co-cultures will evolve with the rapid development of biotechnology in the upcoming years. New drugs that interact with various bone cells have been identified. For example, menaquinone-4, also known as a part of the vitamin K2 family [164] and MSDK (melatonin, strontium, vitamin D3 and vitamin K2) were very recently identified in a dynamic co-culture model of osteoblasts and osteoclasts for being potential substances to reduce osteoporosis [165,166].

Furthermore, humanized IL-18BP has been suggested to be a treatment option for postmenopausal osteoporosis [167]. In the same line of evidence, Janus kinases were proven to inhibit osteoclastogenesis by suppressing RANKL expression in osteoblasts, and may therefore be effective against osteoporosis as well as inflammatory bone diseases [168]. Epimedium, a Chinese traditional medicinal plant extract, has long been used to promote bone tissue growth, and has been further proven to be an efficient treatment against osteoporosis by co-culture models of osteoblasts and osteoclasts [169,170].

It is and will be undoubtedly a future challenge to further optimize co-culture models of osteoblasts and osteoclasts resulting in an easy translation to human beings. However, only if the *in vitro* models are as close as possible to “real” bone, will we hopefully better understand the osteogenesis and/or pathogenesis of several (frequently seen in elderly) bone diseases. However, before addressing possible interactions between osteoblasts and osteoclasts, it is mandatory to succeed with an optimal translation from 2D to 3D co-cultures. Three-dimensional scaffolds seem the most promising option for combining osteoblasts and osteoclasts at present. However, the ideal properties of the 3D scaffolds, like pore size, porosity, stiffness, nutritional transport and mechanical stimulation still need to be defined. Once these characteristics are identified, optimized 3D printing methods bare the beauty of generating defined 3D scaffolds. In addition, their adaptation to a dynamic bioreactor platform will be very important, as this promises the best translation to the in vivo situation when using human cells [171]. To do so, this includes the constant adaptation, optimization and development of analytic methods. There has been lots of progress in optimizing the sensitivity of existing methods, which can help to overcome the large dilution of factors that often occurs in dynamic 3D cultures. However, there is still a need for a better analysis of cells within a scaffold as well as for a universal normalization method, as many of the used assays interfere with the 3D culture conditions or cannot differentiate between different cell types in a co-culture system.

With the optimized conditions, bone cell co-culture models can be modified to simulate specific bone diseases with attempts that have been done before in 2D mono-cultures, e.g., increasing the concentration of glucose and insulin in the culture medium to simulate a diabetes mellitus [11] or even more personalized replacing FCS in the culture medium or the protein source of the scaffold with patients sera [10]. This way bone cell co-culture models may become a powerful tool to understand pathological changes in metabolic bone diseases to identify novel drug targets. Furthermore, these models can then be used for preclinical drug testing. When a model is fast to perform and if it is reliably using primary human cells, it might be even feasible to test individual therapeutic strategies.

## Figures and Tables

**Table 1:** Co-culture models in 2D.

	Figure	Advantages	Disadvantages	Ref.
**conditioned medium**	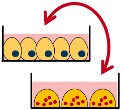	simple setup established methods may be used easy to quantify metabolic and functional changes of the different cells	no direct cell-cell contact medium has to be carefully selected	[51,52,53]
**transwell co-culture**	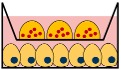	used to investigate paracrine signaling and response to soluble signaling factors cells can be tested separately can be used to analyze cell migration	no direct cell-cell contact medium has to be carefully optimized large volumes needed might limit the use of sensitivity-based methods large volumes needed might limit the oxygen supply in the bottom wells	[54,55,56,57,58]
**removable permeable divider**	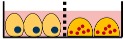	used to investigate paracrine signaling and response to soluble signaling factors cells can be tested separately requires smaller volumes than in the transwell co-culture (assay sensitivity) same medium height (oxygen supply) for both cell types	no direct cell-cell contact medium has to be carefully selected only immature dividers available unknown origin of secreted factors	[49]
**direct co-culture**	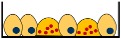	simple setup allows cell-cell contact partly mimics the in vivo situation requires smaller volumes than in the transwell co-culture (assay sensitivity) same medium height (oxygen supply) for both cell types	cell ratios have to be optimized medium has to be carefully selected limited amount of methods available to analyze cells separately unknown origin of secreted factors	[47,48,59,60]

Red arrow: transfer of (conditioned) culture medium.

**Table 2:** Co-culture models in 3D.

Category		Description	Advantages	Disadvantages	Ref.
**hydrogels**	natural hydrogel	water-swollen and cross-linked polymer network made from naturally occurring monomers	provides natural ligands long history of application may contain growth factors	ingredients may be animal-derived inter-assay variation supply of nutrients/oxygen depends on diffusion dead cells get trapped inside	[64,65,66]
	synthetic hydrogel	water-swollen and cross-linked polymer network from synthetic monomers	not animal-derived little inter-assay variation may be functionalized by adding supplements	supply of nutrients/oxygen depends on diffusion dead cells get trapped inside growth factors need to be added upon demand	
**cell aggregation**	hanging drop plates	special plates that allow consistent and controllable cell-aggregation into 3D spheroids	the amount of medium and size of spheroids is controllable mimics anaerobic cond. in tumors flexible combination of cell types small volume (assay sensitivity)	partial oxygen pressure is very high no medium change possible agglomeration is cell dependent supply of nutrients/oxygen depends on diffusion dead cells get trapped inside	[68,69,70]
	3D petri dish	molds made of agarose that favor agglomeration of cells in a defined shape	defined volume within the agarose mold keeps spheroids hydrated mimics anaerobic cond. in tumors flexible combination of cell types medium can be changed	agglomeration is cell dependent supply of nutrients/oxygen depends on diffusion dead cells get trapped inside	
**scaffolds**	natural scaffolds	3D (polymer) matrix made of naturally occurring extracellular matrix	provides natural ligands 3D structures can be adapted to mimic well the natural situation may contain growth factors	ingredients may be animal-derived inter-assay variation supply of nutrients/oxygen depends on diffusion limited ingrowth depth	[71,72,73]
	synthetic scaffolds	3D (polymer) matrix made of different synthetic materials	not animal-derived little inter-assay variation 3D structures can be adapted to mimic well the natural situation may be functionalized by adding supplements	supply of nutrients/oxygen depends on diffusion limited ingrowth depth	
**dynamic models**		often a 3D culture within a bioreactor to provide medium exchange and/or mechanical stimulation	continuous medium exchange provides good supply of nutrients/oxygen for long term culture scaffolds can be chosen based on the requirements mechanical stimulation can be added to the culture	often large volumes of medium required (sensitivity of assays) requires a scaffold/limited perfusability of hydrogels often very complex/only limited amount of experiments can be performed at the same time	[74,75]

**Table 3:** Cell types used in co-cultures for bone metabolism.

Cell Types		Advantages	Disadvantages	Ref.
**osteoblastic lineage**	primary animal osteoblasts and MSCs	no cancerous or transformed characteristics convenient management of donors good reproducibility	use of animals (limited cell availability) species dependent alterations inbred strains cannot represent the existing donor variability	[118,119]
	primary human osteoblasts and MSCs	no cancerous or transformed characteristics no species dependent differences/represent best the human in vivo situation show the existing donor variability	large donor differences (reproducibility?) limited amount of cells experiments have to be performed upon donor availability	[59,109,125]
	osteogenic cell lines	convenient maintenance unlimited cell numbers very good reproducibility different cell lines exist that represent the differentiation states of osteogenic cells relative phenotypic stability	often of cancerous origin or genetically transformed (immortalized primary cells) unable to represent primary cells completely cell lines cannot represent the existing donor variability	[120,121,123,124]
**osteoclastic lineage**	human induced pluripotent stem cells (hiPSCs)	unlimited cell numbers can be generated donor specific	genetically transformed cells reproducibility between different donors? efficiency of the differentiation (osteogenic and osteoclastogenic) protocols in co-culture?	[114,115]
	osteoclasts derived from animal monocytes	no cancerous or transformed characteristics convenient management of donors good reproducibility	use of animals (limited cell availability) species dependent alterations require cytokine cocktails for differentiation inbred stains cannot represent the existing donor variability	[118,119]
	osteoclasts derived from human monocytes	no cancerous or transformed characteristics no species dependent differences/represent best the human in vivo situation show the existing donor variability enough cells can be obtained from minimal invasive blood sampling	large donor differences (reproducibility?) require cytokine cocktails for differentiation experiments have to be performed upon donor availability	[59,109,125]
	osteoclasts derived from myeolytic (monocyte- or macrophage-like) cell lines	convenient maintenance/no time-consuming isolation procedures unlimited cell numbers very good reproducibility relative phenotypic stability	often of cancerous origin unable to represent primary cells completely existing cell lines show different osteoclastic capacity–need to be carefully chosen cannot represent the existing donor variability	[120,121,123,124]

**Table 4:** Methods to characterize and normalize osteoblast and osteoclast cultures.

Visual/Microscopic Methods			
Methods	Use	Limitations	Ref.
van Gieson staining	histological method using picric acid and acid fuchsin to detect collagen	detection is limited by the light transmission of the matrix 3D matrices require thin sectioning (embedding problem) 3D matrices, containing collagen, give false-positive results	[128,129,130]
von Kossa staining	silver ions react with phosphates to demonstrate calcium phosphates	detection is limited by the light transmission of the matrix 3D matrices require thin sectioning (embedding problem) 3D matrices with hydroxyapatite give false-positive results	[59,114,131,132]
Alizarin Red staining	An anthraquinone dye to detect presence of calcium ions. Resolving the stain enables its quantification	detection is limited by the light transmission of the matrix 3D matrices require thin sectioning (embedding problem) 3D matrices containing calcium (e.g., hydroxyapatite, calcium carbonate, etc.) give false-positive results resolved stain might be trapped within a 3D matrix	[59,128,133]
TRAP staining	staining substrate (colorimetric or fluorogenic) is converted by TRAP to identify osteoclasts	detection is limited by the light transmission of the matrix (confocal microscopy can be used to visualize fluorescent stained cells up to a limited depth) 3D matrices require thin sectioning (embedding problem) fixation and embedding procedure affects enzyme activity required for the staining	[134,135]
AP staining	staining substrate (colorimetric or fluorogenic) is converted by AP to identify differentiating osteoblasts		[135]
Pit assay	staining of resorption pits left by osteoclast on dentine chips. Shows bone resorption activity	3D matrices with uneven surfaces mineralization cannot be used detection method (stain of remaining matrix or remaining cells) needs to be carefully chosen, based on the available microscope	[136]
SEM images	SEM can be used to analyze morphological characteristic of cells and 3D matrices	samples must not contain water–drying might affect morphology/cannot be achieved with embedded samples method can only be used up to a certain depth (surface images)	[137,138]
SRB staining	SRB binds to protonated amino- acids, which can be used to quantify total protein content and thus to determine the cell density	staining cannot differentiate between osteoblasts and osteoclasts in a co-culture system 3D matrices containing proteins (e.g., collagen, fibronectin, etc.) give false-positive results	[59,139]
nuclear staining	A large variety of colorimetric (trypan blue, hematoxylin, etc.) and fluorescent (DAPI, Hoechst 33342, propidium iodide, SYTOX green, ethidium homodimer, etc.)	staining cannot differentiate between osteoblasts and osteoclasts in a co-culture system 3D matrices require thin sectioning (embedding problem) stain needs to be carefully chosen (3D matrices might have interfering autofluorescence) based on the available microscope	[140,141]
**Functional Assays**			
**Methods**	**Descriptions**	**Limitations**	**Ref.**
AP activity	colorimetric or fluorogenic substrate is converted by osteoblastic AP	formed products might be trapped within a 3D matrix transport of the substrate/product is dependent on diffusion in a 3D matrix when the culture volume needs to be increased, the sensitivity of the assays might be strongly reduced	[138,142]
TRAP activity	colorimetric or fluorogenic substrate is converted by osteoclastic TRAP		[143,144]
CTSK activity	colorimetric or fluorogenic substrate is converted by osteoclastic CTSK		[145]
CAII activity	colorimetric or fluorogenic substrate is converted by osteoclastic CAII		[146,147]
zymo- graphy	method to detect proteolytic enzymes, which get separated by non-denaturing gel electrophoresis	proteolytic enzymes might be trapped within a 3D matrix their transport is dependent on diffusion out of a 3D matrix reduced sensitivity with increasing culture volume	[148,149]
MTT/XTT assay	tetrazolium salts are reduced in mitochondria of viable cells (toxic), often used for normalization	staining cannot differentiate between osteoblasts and osteoclasts in a co-culture system metabolic activity of a cell might be altered by the stiffness of a 3D matrix (usually higher metabolism on stiffer matrix) stain/product might be trapped within a 3D matrix	[150]
Resazurin conversion	Resazurin (non-toxic) is reduced to the Resorufin in viable cells, which is often used for normalization		[151]
LDH activity	LDH enzyme, located in the cytosol of most cells, leaks into the culture supernatant when cell membranes get damaged	released LDH might be trapped within a 3D matrix detection method needs to be carefully chosen based on task (LDH in the culture supernatant for quantifying damaged cells/LDH in cell lysates for quantifying the remaining cells)	[105,152]
**Immunological Methods**			
**Methods**	**Descriptions**	**Limitations**	**Ref.**
flow cytometry	cells in a single cells suspension can be characterized based on their size, granularity and chosen stain (see figure on molecular markers)	method depends on the isolation of intact cells from a 3D matrix (limitations based on the scaffolds permeability; particles may be released from the 3D matrix interfering with the detection)	[110]
Enzyme-Linked Immuno-Sorbent Assays	ELISA are used to quantify soluble target proteins, e.g., PICP; PINP; DPD; Pi; CTX; NTX; PYD; sclerostin; DKK1&2; MMPs; TIMPs; osteocalcin	target proteins might be trapped within a 3D matrix their transport is dependent on diffusion out of a 3D matrix reduced sensitivity with increasing culture volume	[44,110]
immuno-stainings	antibodies are used to detect target proteins (see figure on molecular markers) in fixed cells; secondary antibodies for detection are either HRP- or fluorophore-labeled	detection is limited by the light transmission of the matrix (confocal microscopy can be used up to a limited depth) 3D matrices require thin sectioning (embedding problem) label of the secondary antibodies has to be carefully chosen (auto-fluorescence of 3D matrices might interfere with fluorophores)	[153]
**Radiological Methods**			
**Methods**	**Descriptions**	**Limitations**	**Ref.**
X-ray images	2D overview image visualizing the mineralized matrix, without destroying the original object	to obtain high resolution images, high X-ray doses have to be applied (affects cell function) can only be used as end point measure, when samples have to be removed from the sterile culture environment markers to detect specific cell function need to be established, which can be taken up from the cells during cell culture	[154]
(micro-)CT images	visualization of mineralized matrix, without destroying the original object, in quantitative 3D images		[155,156,157]
PET-CT images	combination of the (micro-)CT technique with specific markers		[156,157]
**Stiffness Measurements**			
**Methods**	**Descriptions**	**Limitations**	**Ref.**
compression tests	mechanical tests to determine the stiffness of a 3D matrix, which changes when cells produce/incorporate matrix during culture	requires a minimum height (min. 6 mm) for measurement, which in turn limits supply of nutrients/oxygen for the cells can only be used as end point measure, when samples have to be removed from the sterile culture environment methods require a plane surface	[158]
atomic force microscopy			[159,160]

AP—alkaline phosphatase; TRAP—tartrate resistant acidic phosphatase; SRB—sulforhodamine B; DAPI—4′,6-diamidino-2-phenylindole; SEM—scanning electron microscopy; CTSK—cathepsin K; CAII—carbonic anhydrase II; MTT—3-(4,5-dimethylthiazol-2-yl)-2,5-diphenyltetrazolium bromide; XTT—2,3-Bis-(2-Methoxy-4-Nitro-5-Sulfophenyl)- 2H-Tetrazolium-5-Carboxanilide; LDH—lactate dehydrogenase; PICP—type 1 procollagen C-terminal propeptide; PINP—Procollagen type I N-terminal propeptide; DPD—deoxypyridinoline; Pi—inorganic phosphate; CTX—C-terminal telopeptide of type 1 collagen; NTX—cross-linked N-telopeptides of type I collagen; PYD—pyridinoline; DKK1 & 2—Dickkopf 1 & 2; MMPs—matrix metalloproteinases; TIMPs—tissue inhibitors for matrix metalloproteinases; HRP—horseradish peroxidase; CT—computer tomography; PET-CT—positron emission tomography-computed tomography.

## Abbreviations

Ad-MSCs	MSCs derived from adipose tissue
AP	alkaline phosphatase
ATF4	activating transcription factor 4
BCL-2	B-cell lymphoma 2
BMP	bone morphogenetic proteins
B-MSCs	MSCs derived from bone marrow
BSP	bone sialoprotein
CAII	carbonic anhydrase II
CALCR	calcitonin receptor
cAMP	cyclic adenosine monophosphate
CD	cluster of differentiation
COL1A1	collagen type I α 1
CT	computed tomography
CTGF	connective tissue growth factor
CTSK	cathepsin K
CTX	collagen type 1 C-telopeptide
DKK1&2	dickkopf 1 & 2
DMP1	dentin matrix acidic phosphoprotein 1
DOK3	downstream of kinase 3
DPD	deoxypyridinoline
ECM	extracellular matrix
FGF-23	fibroblast growth factor 23
HGF	hepatic growth factor
hiPSCs	human induced pluripotent stem cells
IGF	insulin-like growth factor
IL	interleukin
IP_3_	inositol trisphosphate
JAK	Janus kinase
LDH	lactate dehydrogenase
MATF	melanogenesis associated transcription factor
M-CSF	macrophage colony stimulating factor
MMP	matrix metalloproteinase
MSCs	mesenchymal stem cells
MSDK	melatonin, strontium, vitamin D3 and vitamin K2
NFATC1	nuclear factor of activated T-cells, cytoplasmic 1
NSAID	nonsteroidal anti-inflammatory drug
OC	osteocalcin
OPG	osteoprotegerin
OSCAR	osteoclast-associated receptor
PBMCs	peripheral blood monocytes
PDGF	platelet derived growth factor
PET-CT	positron emission tomography-computed tomography
PICP	procollagen type I carboxy-terminal propeptide
PINP	procollagen type I N-terminal propeptide
RANK	receptor activator of nuclear factor-kb
RANKL	receptor activator of nuclear factor-kb ligand
RUNX2	runt-related transcription factor 2
S1P	sphingosine-1-phosphate
SATB2	special AT-rich sequence-binding protein 2
SEM	scanning electron microscopy
SFRP1	secreted frizzled related protein 1
SOST	gene name for sclerostin
Sphk1	sphingosine kinase 1
SRB	sulforhodamine B
TGF-β	transforming growth factor beta
THPO	thrombopoietin
TRACER	tissue roll for the analysis of cellular environment and response
TRAF6	TNF receptor associated factor 6
TRAP	tartrate-resistant acid phosphatase 5b
VitDR	vitamin D receptor
